# Psychobiological Evaluation of Day Clinic Treatment for People Living With Dementia – Feasibility and Pilot Analyses

**DOI:** 10.3389/fnagi.2022.866437

**Published:** 2022-06-30

**Authors:** Alexandra Wuttke-Linnemann, Svenja Palm, Katharina Geschke, Nadine Skoluda, Theresa Bischoff, Urs M. Nater, Kristina Endres, Andreas Fellgiebel

**Affiliations:** ^1^Center for Mental Health in Old Age, Landeskrankenhaus (AöR), Mainz, Germany; ^2^Department of Psychiatry and Psychotherapy, University Medical Center, Johannes Gutenberg-University Mainz, Mainz, Germany; ^3^Department of Clinical and Health Psychology, University of Vienna, Vienna, Austria; ^4^University Research Platform “The Stress of Life (SOLE) – Processes and Mechanisms Underlying Everyday Life Stress”, University of Vienna, Vienna, Austria; ^5^Hospital for Psychiatry, Psychosomatic and Psychotherapy, Agaplesion Elisabethenstift, Darmstadt, Germany

**Keywords:** aging, autonomic nervous system, HPA axis, neurodegeneration, psychophysiology, psychiatric day unit, geriatric psychiatry, memory clinic

## Abstract

**Background:**

Hospitalization is often stressful and burdensome for people living with dementia (PwD) and their informal caregivers (ICs). Day clinic treatment may provide a suitable alternative, but is often precluded by a diagnosis of dementia. Furthermore, it is often caregiver-based ratings that measure treatment success as the validity of self-reports in PwD is critically discussed. We therefore set out to examine the feasibility of psychobiological stress measures in PwD and ICs and to evaluate treatment trajectories considering both the day clinic context and the daily life of the dyads.

**Method:**

A total of 40 dyads of PwD (mean age: 78.15 ± 6.80) and their ICs (mean age: 63.85 ± 13.09) completed paper-and-pencil questionnaires (covering stress, depressive symptoms, and caregiver burden among others) in addition to the measurement of hair cortisol concentrations (HCC) at admission, discharge, and follow-up 6 months after day clinic treatment. As part of an ambulatory assessment, for 2 days at the beginning and 2 days at the end of the day clinic treatment, PwD and ICs collected six saliva samples per day for the analysis of salivary cortisol (sCort) and alpha-amylase (sAA).

**Results:**

Paper-and-pencil questionnaires and HCC assessments were more feasible than the ambulatory assessment. We found discrepancies between subjective and physiological markers of stress in PwD. Whereas HCC decreased over time, self-reported stress increased. Child–parent dyads reported decreases in neuropsychiatric symptoms, associated burden, and self-reported stress from admission to follow-up. In daily life, both PwD and ICs showed characteristic diurnal profiles of sAA and sCort, however, we found no differences in summary indicators of salivary stress markers over time.

**Discussion:**

The psychobiological evaluation was feasible and added informative value, underlining the potential of physiological stress markers to complement self-reports on stress in PwD and to objectively evaluate treatment trajectories. In this sample, HCC was more feasible and acceptable as biological marker of stress compared to saliva samples. Concerning treatment trajectories, differential effects on the dyads were found, with child–parent dyads benefiting more from day clinic treatment compared to spousal dyads.

## Introduction

### Stress in People Living With Dementia and Their Informal Caregivers

Stress, and particularly chronic stress, has harmful effects on people living with dementia (PwD) and their informal caregivers (ICs). Adverse health effects are reported in both, with chronic stress leading to faster disease progression in PwD (Csernansky et al., 2006) and diminished health in their ICs (Bauer et al., 2000). From a dyadic perspective, however, stress not only affects the individual, but rather affects both members of the dyad (Wuttke-Linnemann et al., 2019). For PwD and their ICs, this dyadic interplay can result in toxic exacerbations and escalations: A common source of stress for ICs is the presentation of behavioral and psychological symptoms of dementia (BPSD) in PwD (e.g., hallucinations, delusions, sleep disturbances, and aggression), which challenge ICs’ resources (Feast et al., 2016). Often, ICs do not have adequate means to meet the needs underlying the BPSD, resulting in further manifestations of and deteriorations in BPSD, and in turn leading to additional stress in ICs. Thus, PwD and ICs are trapped in a vicious cycle that frequently leads to poor outcomes for both parties: Caregivers often present with fatigue and exhaustion, reduced quality of life, increased depression, poorer health, and lower income (Kales et al., 2015; Feast et al., 2016); PwD experience higher rates of emergency hospital admissions and disruptions in care, early admission to nursing homes, faster disease progression, and increased morbidity and mortality (Kales et al., 2015).

#### (Emergency) Hospital Admissions and Disruptions in Care

PwD are more often admitted to hospital than age-matched patients without dementia (Joyce et al., 2007; Frytak et al., 2008), and BPSD, associated with high caregiving burden, place PwD at a particularly increased risk of hospitalization (Toot et al., 2013). However, these hospital admissions have a series of adverse health outcomes. Compared to older adults without dementia, hospitalization of PwD is accompanied by increased rates of complications, including a higher prevalence of delirium, dehydration, and pain, worsened cognitive and physical status, increased behavioral and psychological symptoms, as well as increased morbidity (e.g., urinary tract infections, decubitus, pneumonia, metabolic imbalance, sepsis, heart failure, myocardial infarction, anemia, complications after surgery, and thrombotic events) and mortality (Morrison and Siu, 2000; Robinson et al., 2009; Sampson et al., 2009; Ehlenbach et al., 2010; Wilson et al., 2012; Bail et al., 2013, 2015; Rao et al., 2016; Hessler et al., 2018). Moreover, PwD have longer hospital stays (Zekry et al., 2009; Mukadam and Sampson, 2011; Motzek et al., 2017), more readmissions, and more transfers to nursing home care (Draper et al., 2011). Nevertheless, about a quarter of hospital admissions of cognitively impaired older adults are caused by ambulatory care-sensitive conditions (Wolf et al., 2019), which are primarily treatable on an ambulatory basis. This implies that improved ambulatory care might reduce the frequency of hospitalizations, which is of particular importance in cognitively impaired older persons.

### Day Clinic Treatment as an Alternative

Hospital treatment should be adapted to the needs of PwD in order to reduce the negative consequences of a hospital stay. Day clinic treatment might therefore present a possible alternative to maintain as much daily routine for the PwD as possible while allowing for as much recovery as possible for the ICs. Although day clinic treatment for psycho-geriatric patients in Germany began in 1976 (Wächtler et al., 1994), it still does not play a significant role in the health care system for these patients in Germany. So far, the number of empirical studies on psychiatric day clinics for PwD remains limited.

There is empirical evidence that interprofessional specific programs for PwD in a day clinic lead to a clear improvement in behavioral symptoms and positively influence the distress of caring relatives (Johansson and Gustafson, 1996; Hoe et al., 2005; Weber et al., 2009; Wunner et al., 2020). Furthermore, day clinic treatment of PwD was found to reduce the 1-year hospital readmission risk, as compared to an increased risk among inpatients (Steinkamp and Werner, 1998; van de Vorst et al., 2018). In a previous study by our work group, we compared sociodemographic and clinical characteristics of voluntarily treated PwD and their ICs between an inpatient setting and a day clinic setting (Linnemann et al., 2018). PwD did not substantially differ in these characteristics between the two settings and the treatment effects were similar. However, concerning ICs, there were significant differences between the two settings. ICs of day clinic patients were significantly older, showed a higher burden due to practical caring responsibilities, lower physical health, and a higher rate of depressive syndromes at follow-up compared to caregivers of inpatients.

While the aforementioned study provided evidence that day clinic treatment is feasible in PwD, it did not address specific research questions. First, all assessment instruments were based on self-report for ICs and on informant-based ratings for PwD. Thus, the perspective of the PwD was not directly included. Second, although stress often endangers the stability of care, leading to hospital admissions, the study did not assess stress *per se*.

### Psychobiological Stress Markers in People Living With Dementia – Need to Complement Subjective Reports With Physiological Markers

The assessment of physiological stress markers allows complementing the subjective perspective of PwD (Wuttke-Linnemann et al., 2019, 2022). Particularly given the questionable validity of self-reports in PwD that may arise due to cognitive impairments resulting in anosognosia (Wilson et al., 2016), physiological stress markers might enable the psychobiological stress experience to be captured more comprehensively. In this regard, we recently found discrepancies between subjective and physiological markers of stress in PwD when evaluating the treatment success of a dyadic home-based psychosocial intervention. Whereas subjective stress did not decrease over time, PwD reported lower secretion of cortisol after each home visit (Wuttke-Linnemann et al., 2022).

While elevated levels of the stress hormone cortisol have been identified as risk factor for the development of dementia (Ouanes and Popp, 2019) and for a faster disease progression in PwD (Csernansky et al., 2006), physiological stress markers are seldom used to evaluate treatment effects in PwD. However, this would be particularly relevant in PwD as the hippocampus is sensitive to chronic stress and glucocorticoids (Conrad, 2008) facilitating further neurodegeneration. Chronic stress has deleterious and neurotoxic effects on the brain with dysregulations in glucocorticoids increasing allostatic load (McEwen, 2001). These adverse health effects of stress on health are mediated by changes in the stress-sensitive systems of the body (McEwen, 1998). Chronic stress increases allostatic load mediated by dysfunctions and dysregulations in the hypothalamic-pituitary-adrenal axis and the autonomic nervous system. This has particular relevance as these stress-sensitive systems interact with the immune system and thus shape the organism’s response to adversity in daily life. However, although the assessment of physiological stress markers holds the potential to inform diagnosis and treatment of PwD, they are seldomly used in PwD and their ICs.

In a feasibility study of salivary cortisol as objective measure for physiological stress in nursing home residents with dementia, Pu et al. (2020) report multiple challenges and high number of missing values most often due to cognitive impairments of the PwD. Hair cortisol might be a promising marker in this regard, as it can be assessed unintrusively and retrospectively captures long-term cortisol secretion. Particularly in older age, the potential of hair cortisol to assess cortisol as a risk factor and as a biomarker to evaluate the effectiveness of stress reduction interventions has been discussed (Wright et al., 2015). However, so far, physiological stress markers such as hair cortisol have most often been assessed in caregivers of PwD rather than directly in PwD (Stalder et al., 2014; Rippon et al., 2021). Empirical evidence on intervention effects as captured by physiological stress markers in PwD is in its beginning. The empirical evidence so far covers a wide and heterogenous range of interventions such as dyadic psychosocial intervention (Wuttke-Linnemann et al., 2022), hand massage (Schaub et al., 2018), acupressure (Kwan et al., 2017), touch (Woods et al., 2009), robot companions (Liang et al., 2017), art interventions (D’Cunha et al., 2019), music therapy (Chu et al., 2014; de la Rubia Ortí et al., 2018), dance therapy (Ho et al., 2020), or exercise (Venturelli et al., 2016). Most often salivary cortisol is assessed as outcome measure with only few studies that simultaneously assessed salivary cortisol and salivary alpha-amylase (Schaub et al., 2018; Wuttke-Linnemann et al., 2022). Methodological aspects vary among these studies considering the study population (severity of dementia), the setting of the study (hospital, nursing home, and home), the number of saliva samples obtained, sampling pattern and collection method, thus limiting comparisons across studies. Nevertheless, in the majority studies point to beneficial effects on salivary cortisol, although often challenges with collecting sufficient valid saliva samples are reported (Kwan et al., 2016). Beneficial effects present themselves by pre to post differences showing decreases in salivary cortisol after participation in a dyadic psychosocial intervention (Wuttke-Linnemann et al., 2022), acupressure (Schaub et al., 2018), hand massage (Kwan et al., 2017), and exercise- and cognitive-based treatments (Venturelli et al., 2016). Whereas one study finds decreases in salivary cortisol after music therapy (de la Rubia Ortí et al., 2018), another one did not (Chu et al., 2014). Also beneficial effects on diurnal rhythms are reported with increases in characteristic markers such as morning-to-evening ratio (D’Cunha et al., 2019) and slope (Ho et al., 2020) and decreases in total daily output as measures by area-under-the-curve (Wuttke-Linnemann et al., 2022).

### Effects Beyond the Clinic Setting – Ambulatory Assessment to Capture Daily Life Experiences

A study by Fonareva et al. (2012) showed that stress ratings differed between research center and home environments, rendering it necessary to assess the effects of an intervention in daily life as well. However, whereas the research center environment has the advantage of monitoring the assessments more closely, control mechanisms cannot be implemented so easily in home environments. In particular, the collection of saliva samples might be less feasible at home than in a research center. Hodgson and Granger (2013) presented recommendations on how to assess saliva samples in older frail patients using the caregivers’ assistance and encouraged to assess these markers in this vulnerable population.

Overall, the empirical evidence points to the dilemma that PwD are often admitted to hospitals even though hospitalization has adverse health effects on them. Alternatives such as day clinic treatment are available, but the evaluation of these alternatives is most often based on informant-based ratings rather than self-report. We therefore set out, to evaluate the feasibility of psychobiological stress markers in both PwD and ICs in the day clinic context as well the home environment by means of an ambulatory assessment approach. Furthermore, we then evaluated the effectiveness of day clinic treatment and treatment trajectories concerning both PwD and ICs over time (admission, discharge, six months follow-up).

## Materials and Methods

### Procedure

The study took place in a day clinic for PwD located in Munich, Germany. Prior to elective admission to the day clinic, all PwD and ICs were informed about the possibility to participate in a scientific evaluation of the day clinic treatment. Inclusion criteria for ICs were age ≥18 years, fluency in the German language, role as the primary informal caregiver, regular contact with the PwD (at least twice a week), and no cognitive impairment (MMSE ≥ 24). Inclusion criteria for PwD were fluency in the German language and a firm or suspected diagnosis of dementia. Concerning the assessment of salivary stress markers, further exclusion criteria were defined for both PwD and ICs: intake of any medication with an effect on the neuroendocrine system, chronic disease affecting the neuroendocrine system, psychiatric condition (substance dependence, psychosis), smoking, body mass index (BMI) ≥ 30, hair shorter than 1 cm. However, to encourage as many dyads as possible to participate in the study, the measurement of physiological stress markers (i.e., saliva and hair samples) was optional.

PwD and their ICs who expressed an interest in participating were informed about the study in a personal meeting, which was scheduled within the first 5 days after admission. It was stressed that participation was voluntary and would not affect treatment in the day clinic. Written informed consent was obtained from both PwD and ICs before participation. PwD with legal guardians were only included if they had basic cognitive capacity and if implicit intentional behavior to participate in the study was shown.

**FIGURE 1:**
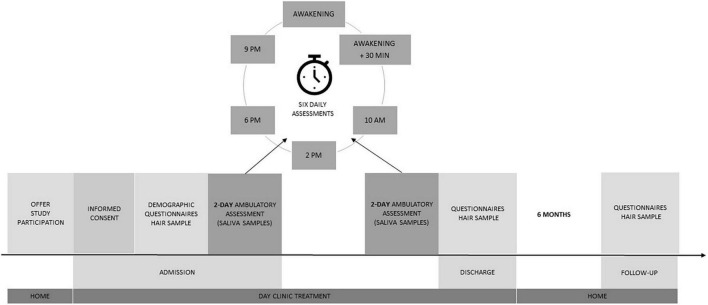
Study procedure.

After inclusion in the study, baseline sociodemographic variables were collected by the study personnel, who also performed the Mini-Mental State Examination (MMSE; Folstein et al., 1983) with the PwD. Next, a hair sample was taken from both dyad members by trained study personnel. PwD and ICs were then asked to complete questionnaires in the subsequent 5 days. If PwD did not have the mental capacity to complete the questionnaires, study personnel were available to provide support. We specifically asked caregivers to refrain from assistance in order to prevent biases. As part of the ambulatory assessment, on the 2 days after study inclusion, subjective stress ratings were gathered and saliva sampling took place, consisting of six daily assessments (awakening, 30 min after awakening, 10 am, 2 pm, 6 pm, and 9 pm) for the analysis of salivary cortisol (sCort) and alpha-amylase (sAA). Directly before discharge, PwD and ICs were asked to complete questionnaires again and complete ambulatory assessment on the 2 days before discharge from the day clinic. Hair samples were retaken at discharge. After 6 months, paper-and-pencil questionnaires were sent out by mail, and both PwD and ICs were invited to an outpatient session at the clinic, where a third hair sample was taken from both dyad members. The study procedure is illustrated in Figure 1. The ethics committees of the Landesärztekammer Bayern (as the day clinic was situated in Munich) and the Landesärztekammer Rheinland-Pfalz (as the evaluation was coordinated in Mainz) approved the study protocol.

### Therapeutic Rationale of the Day Clinic

The day clinic offers 20 outpatient places for patients with memory disorders and dementia. Only voluntarily treated patients can be electively admitted, as there are no closed wards. Treatment and travel costs (transportation by a special driving service) are usually covered by health insurance companies. At night and on weekends, patients are at home. Support is offered in organizing assistance or planning a care concept for the time at night and weekends. Based on guideline-oriented diagnostics and treatment, a holistic approach to treatment is taken, focusing on the needs of the individual patient. In most cases, treatment is scheduled to last 4–6 weeks. During this time, the patients take part in various therapeutic offers from Monday to Friday, according to an individual therapy plan. Treatment is scheduled workdays from 8.30 am to 5.00 pm (Fridays 8.30 am to 4.00 pm). A variety of non-pharmacological treatments are offered, such as music therapy, occupational therapy, art therapy, movement therapy and psychotherapy among others. Additionally, the patients also receive an individually tailored drug treatment plan according to guidelines. Further, one focus of the day clinic is the inclusion of the ICs and the social environment in order to strengthen the sustainability of the therapy.

### Assessment Instruments

#### Psychometric Test Battery

PwD and ICs completed the following paper-and-pencil scales at each assessment point (admission, discharge, follow-up):

To assess depressive symptoms, we used the short form of the Geriatric Depression Scale (GDS-15) (Sheikh and Yesavage, 1986). The GDS-15 is validated for older populations with an MMSE score of 10 or more (Conradsson et al., 2013) and is also regularly used for the assessment of depressive symptoms in caregivers of PwD (Covinsky et al., 2003). The scale comprises 15 items rated using a dichotomous response format (yes/no). After recoding five reverse-coded items, a sum score is calculated, with sum scores ≥5 indicating clinically relevant depressive symptoms.

Subjectively perceived stress levels were assessed using the Perceived Stress Scale (PSS-10, Cohen, 1988). Chwalisz and Kisler (1995) found the PSS to be particularly suitable in the context of caregiving compared to traditional measures on caregiver burden. Deeken et al. (2018) demonstrated good psychometric properties of the PSS in both PwD and ICs. Participants are asked to rate ten items on a scale ranging from 0 (never) to 4 (often) referring to the past 4 weeks. After recoding four reverse-coded items, a sum score is calculated, with higher scores representing higher stress.

We used the Screening Scale for Chronic Stress (SSCS) from the Trier Inventory for the Assessment of Chronic Stress (TICS) (Schulz et al., 2004). The SSCS is a summary scale based on the 12 items that loaded highest on the first factor in the validation of the TICS. It comprises items from five different types of stress: sorrow, work overload, social stress, work discontent, and lack of social recognition. Higher sum scores indicate higher chronic stress.

The German version of the Brief Resilience Scale (BRS) was used to measure trait resilience (Chmitorz et al., 2018). The scale comprises six items rated on a scale ranging from 1 (strongly disagree) to 5 (strongly agree). After recoding reverse-coded items, a mean score is calculated.

Additionally, ICs were asked to rate BPSD and functional independence of the PwD at all three assessment points:

To assess behavioral symptoms of dementia, we used the 12-item version of the Neuropsychiatric Inventory (NPI) (Cummings et al., 1994). In this interview, caregivers are asked to rate the occurrence of 12 domains of behavioral symptoms (e.g., delusions, hallucinations, depression, anxiety, and sleep disturbances). If any of the domains occur, caregivers are asked to rate the frequency and severity of each symptom on a scale ranging from 1 to 4 for frequency (1 = occasionally, less than once per week to 4 = very frequently) and 1 to 3 for severity (1 = mild, 2 = moderate, 3 = severe). A total score is calculated as the sum of the product frequency*severity, which can vary between 0 and 144, with higher scores indicating higher frequency and severity of BPSD. We further included the Caregiver Distress Scale (Kaufer et al., 1998), on which caregivers are asked to rate their distress on a scale ranging from 0 (=no distress) to 5 (=very severe or extreme stress) for each confirmed domain. Summing this distress rating for each domain, one can calculate the NPI caregiver distress score, which can vary between 0 and 60.

The Bayer Activities of Daily Living Scale (B-ADL) is an informant-rated scale on impairments in activities of daily living in older people with cognitive impairments (Hindmarch et al., 1998). With a total of 25 items, informants are asked to rate the frequency of problems in everyday life functioning on a 10-point scale ranging from 1 (never) to 10 (always), with higher scores thus indicating higher impairments in activities of daily living.

The Barthel index is an informant-rated scale on independence in activities of daily living (Mahoney and Barthel, 1965), thus indicating the degree of care needs. A total of 10 items are rated, with higher scores indicating higher autonomy.

#### Physiological Stress Markers

Hair cortisol concentrations (HCC): One to three hair strands were cut as close as possible to the scalp from the posterior vertex region of the head by trained study personnel. For determination of HCC, the first proximal 2 cm segment was used which is thought to reflect the cumulative cortisol secretion of the past 2 months (Wennig, 2000). Hair wash and cortisol extraction procedures based on laboratory protocol by Stalder et al. (2012), with minor modifications. In brief, hair samples were washed twice in a glass vial for 3 min using 3 mL isopropanol. For cortisol extraction, 7.5 ± 0.5 mg whole, finely cut hair were incubated in 1.8 mL methanol in a glass vial. After incubation for 18 h at room temperature, 1.6 mL were transferred in another glass vial. Then, 1.6 mL of the supernatant was evaporated at 50°C until samples were completely dried. Finally, the samples were resuspended with 225 μL ultra-pure water and immediately vortexed for 20 s. For cortisol determination, a commercially available cortisol luminescence immunoassay was used (LIA; IBL International, a Tecan Group Company, Hamburg, Germany). Inter- and intra-assay coefficients of variation were 3.4 and 6.0%, respectively.

The ambulatory assessment combined momentary subjective stress ratings with the measurement of salivary cortisol (sCort) secretion and salivary alpha-amylase (sAA) activity at each time point. Momentary subjective stress was assessed using a one-item approach to keep participant burden to a minimum. Using printed questionnaires, participants were asked to rate the item ‘At this moment, I feel stressed’ on a visual analog scale (VAS) ranging from 0 (‘not at all’) to 100 (‘completely’). Furthermore, at each of the time points, PwD and ICs were asked to transfer accumulated saliva into pre-labeled vials using SaliCaps (IBL International, a Tecan Group Company, Hamburg, Germany). Participants were asked to store the saliva samples as cool as possible at home in their freezer and to bring them to the day clinic at their earliest convenience, whereupon the samples were frozen at −80°C. Additionally, participants were asked to refrain from eating, drinking (except for water or tea without sugar), or intensive physical activity for 1 h prior to sampling. As a compliance check, participants had to document whether they had eaten, drunk or engaged in intensive physical activity prior to saliva sampling. Two of the daily saliva samples of the PwD fell within the time of the day clinic stay (10 am and 2 pm). These two samples were thus collected by nursing staff of the day clinic and directly stored at −80°C. For the remaining saliva samples, ICs were asked to assist the PwD in collection, as recommended by Hodgson and Granger (2013). sCort levels were measured using a commercially available enzyme-linked immunoassay (IBL International, a Tecan Group Company, Hamburg, Germany). sAA activity was measured using a kinetic colorimetric test and reagents obtained from DiaSys Diagnostic Systems (Holzheim, Germany). Inter-assay variance was 13.3% for sAA and 12.6% for sCort, and intra-assay variance was 14.2% for sAA and 1.67% for sCort. Summary indices (area-under-the-curve with respect to ground, AUCg) were calculated according to the formula provided by Pruessner et al. (2003) including the six daily assessments at admission and discharge each. Cortisol awakening response (CAR) was calculated as percentage rise in sCort secretion from awakening to 30 min after awakening.

### Statistical Analysis

Analyses were performed using SPSS (IBM SPSS Statistics for Windows, Version 23.0, IBM Corporation, Armonk, NY, United States). Concerning feasibility, we calculated the amount of missing values per parameter and report reasons for this if applicable. In addition, we tested whether the missing values in our data set were associated with specific characteristics of the sample. We created dummy variables with a 0 (value missing)/1 (value present) coding for HCC and saliva sample measures. We then calculated the correlation with potential influencing variables (Time, Person, Gender, Relationship, MMSE, NPI, and GDS) with these dummy variables. Additionally, we tested further for dependencies among these variables by means of a series of 2 × 2 chi-square tests (person × GDS/PSS/SCSS/BRS; sAA/sCort/CAR/HCC × GDS/PSS/SCSS/BRS; sAA × HCC) separately for each time point (baseline, discharge, and follow-up). Since very small cell abundances were expected in some comparisons, we report the results of the Fisher exact test. Concerning the evaluation of treatment trajectories over time, we estimated multilevel models (MLM) using the MIXED function. Construction of the models and subsequent interpretation was done in accordance with Bolger and Laurenceau (2013). In a stepwise procedure, we first created a base model that solely included the outcome variable (scores from paper-and-pencil questionnaires, HCC, ambulatory assessment data). As the intraclass correlation (ICC) (=the amount of variance between second-level units in relation to the total variance) was >0.20 for all base models, we estimated follow-up MLMs. In these MLMs, we first tested the model for a random effect of the variable Time. If the inclusion of the random effect (Time) did not improve model fit, all further models were calculated with fixed effects only. Concerning the outcome variables Bayer-ADL, NPI Burden and HCC, including Time as random effects variable improved model fit. These random effects models can be found in Supplementary Material A as we report fixed effects models focusing on between-subject differences here in the manuscript. We went on including further predictors into the model in a stepwise manner: 1st: Time, 2nd: Person (IC vs. PwD), 3rd: control variables (Gender; in the case of psychobiological outcome measures we further included age and BMI), 4th: Relationship (spousal dyad vs. child–parent dyad) as fixed effects variables due to known associations with the outcome measures. Comparison between the models and the base model was made by comparing model information criteria. Specifically, we interpreted the Bayesian Information Criterion (BIC), with lower figures representing better model fit. We stopped the inclusion of additional predictors when the model fit was worsened by the inclusion of additional predictors.

## Results

### Participants

A total of 40 dyads of PwD and their IC participated in the study. PwD (17 female) were 78.15 ± 6.80 years old (range: 57–94) and ICs (31 female) were 63.85 ± 13.09 years old (range: 36–84). A total of 39 PwD had already a secure diagnosis of dementia at admission, whereas one patient was diagnosed with a suspected diagnosis of dementia that was confirmed during the day clinic stay. The majority of PwD was diagnosed with Alzheimer’s disase (F00.1, *n* = 22; F00.2, *n* = 8; F00.0, *n* = 1) followed by unspecified dementia (F03, *n* = 4), dementia in other diseases classified elsewhere (F02.3, *n* = 3; F02.0, *n* = 1), and vascular dementia (F01.9, *n* = 1). Dyads were either married couples (59.2%) or child–parent constellations (40.8%). The mean MMSE sum score of PwD was 16.10 ± 6.57. PwD reported that they had suffered from cognitive symptoms for 44.62 ± 34.31 months, ranging from 1 to 132 months. At baseline, a total of 28 PwD had been assessed as requiring care (mean care level: 2.32 ± 1.06, range 0–5). ICs reported a mean of 54.93 ± 61.50 h per week spent on caregiving (range 2.50–168 h). A total of 15 out of 40 ICs (37.5%) reported using support services. Concerning the work situation of the ICs, 50.7% were retired, 39.1% were in employment, 7.2% stayed at home, and 2.9% were unemployed.

### Feasibility Aspects – Missing Values and Completeness

Concerning the paper-and-pencil psychometric test battery, missing values of each test varied between 0 and 12 at baseline resulting in completion rates of 85.00–100.00%. At discharge missing values varied between 6 and 19 per test (76.25–92.50% completion rate), and at follow-up between 6 and 23 (71.25–92.50% completion rate). Completeness of assessment varied between PwD and ICs, with the completeness of assessments of PwD varying between 22 and 40 PwD per test (55.00–100.00%) and completeness of ICs varying between 34 and 39 ICs per test (85.00–97.50%).

With regard to HCC, a total of nine participants (six PwD, three ICs) did not provide hair samples at any of the three time points (11.25%). Another individual 19 hair samples could not be collected. The reason for this was most frequently insufficient amount of hair or lack of possibility to be present at the on-site appointment. No participant reported reservations or objections. Thus, a total of 194 hair samples was sent to the laboratory for analysis. There, a further 17 samples were excluded [too short hair (<2 cm, *n* = 12), insufficient amount of collected hair (*n* = 4), one outlier (>3 SD) in HCC (216.07 pg/mg)]. Thus, a total of 177 (out of 240 possible) hair samples were available for analysis, equaling 73.75% of overall completion. Sixty-four samples were entered into the analysis at baseline, 58 at discharge, and 55 at follow-up. Considering the sub-sample that provided hair samples, completion rates varied between 77.46 and 90.14% at each assessment point. However, only 16 (out of 40) dyads provided hair samples at all three assessment points. Missing values in HCC were correlated with GDS-15 sum score in that higher depression scores were related to missing values (*r* = −0.195, *p* = 0.003).

In terms of the ambulatory assessment, five dyads, three individual ICs, and three individual PwD did not collect saliva samples at all, resulting in the exclusion of 16 out of 80 participants (20.00%) in the respective analyses. Furthermore, three dyads and one individual IC only provided saliva samples at the beginning of day clinic treatment (but not at the end). Thus from a potential of 1,920 saliva samples, only 1,452 saliva samples were possible. Considering this sub-sample that provided saliva samples, a total of 1,139 cortisol values (78.44% completeness) and 1,086 alpha-amylase values (74.79% completeness) were entered into the analysis. Reasons for missing values were either that they could not be collected in daily life (interference with current activity) or that they were excluded in the lab due to insufficient amount of saliva. A total of 9 PwD was not able to collect saliva samples at home and thus only provided those saliva samples that were collected by the day clinic personal during the day clinic stay. Subjective stress ratings were available in 948 cases (65.29% completeness). The area-under-the curve (AUC), as a summary indicator, could only be calculated in the case of six complete assessments per day. This occurred for 114 days concerning VAS, 103 days concerning sCort, and 91 days concerning sAA (out of 256 possible days). Correlation analyses show that missing values for CAR, sCort and sAA occurred more often in PwD (*p* < 0.01), more often at discharge (*p* < 0.01), and were more often in child–parent dyads than spousal dyads (*p* < 0.01). Further, the amount of missing values increase with decreasing MMSE (*p* < 0.01) and increasing GDS-15 sum sore (*p* < 0.05). Results of the correlation analyses can be found in Supplementary Material B. The results of the chi-square tests indicate that there are statistical dependencies between the missing values of the questionnaires and the person (more frequent in PwD) especially at later time points and between missing values of the questionnaires and missing values in the biomarkers also at later time points (Supplementary Material C).

### Feasibility Aspects – Reliability of Paper-and-Pencil Questionnaires

Indicators of reliability concerning the paper-and-pencil questionnaires were at least good in both PwD and ICs. The reliability of GDS-15 was even higher in PwD (α = 0.84, ω = 0.84) than in ICs (α = 0.79, ω = 0.79). Concerning the PSS, reliability was high in both PwD (α = 0.79 and ω = 0.80) and ICs (α = 0.87 and ω = 0.87), as compared to α = 0.84 in the German validation study (Klein et al., 2016). It is of note, that in the present sample, the mean sum score of PSS was 18.13 ± 7.2 in ICs and 15.85 ± 6.99 in PwD compared to normative data from a German validation study in which the mean score on the PSS in the subgroup of participants aged ≥60 years lay at 11.94 ± 6.14 (compared to $\bar{\text{x}}$ = 12.57 in the total sample). Concerning chronic stress levels, reliability was high in ICs (α = 0.93 and ω = 0.94) and in PwD (α = 0.91 and ω = 0.91), as compared to α = 0.91 in the TICS validation study. At baseline, PwD had a mean score of 10.82 ± 9.57 and IC had a mean score of 22.63 ± 10.24, as compared to 14.37 ± 8.22 in the TICS validation study. Concerning BRS, reliability was higher in ICs (α = 0.82; ω = 0.83) than in PwD (α = 0.64, ω = 0.65), as compared to α = 0.85 in the validation study. In the present study, the mean BRS value at baseline was 3.23 ± 0.81 in ICs and 3.63 ± 0.67 in PwD. In a population-based validation study, mean values for the BRS in two general population samples with a mean age of 42.56 ± 26.52 and 51.05 ± 17.90 were 3.58 and 3.37 (Chmitorz et al., 2018). In a study examining effects of a home-based dyadic psychosocial interventions of PwD and ICs, we found mean BRS values at baseline of 3.13 ± 0.78 for ICs and 3.00 ± 0.38 for PwD (Wuttke-Linnemann et al., 2020).

### Clinical Characteristics Over Time – Subjective Ratings

The mean duration of stay in the day clinic was 33.13 ± 10.53 days (including weekends). The mean values of the psychometric test battery and physiological stress markers over time can be found in Table 1.

**TABLE 1:** Results from psychometric test battery over time.

	PwD			IC		
	
	Admission	Discharge	Follow-up	Admission	Discharge	Follow-up
	
	*X* ± *SD* (*n*)	*X* ± *SD* (*n*)	*X* ± *SD* (*n*)	*X* ± *SD* (*n*)	*X* ± *SD* (*n*)	*X* ± *SD* (*n*)
GDS-15	4.80 ± 3.42(40)	3.68 ± 3.56(34)	5.92 ± 3.87(25)	3.85 ± 2.83(34)	4.29 ± 3.07(35)	4.35 ± 3.33(37)
PSS-10	15.27 ± 6.96(37)	14.12 ± 6.26(33)	19.41 ± 7.11(22)	19.10 ± 6.93(39)	17.91 ± 6.88(35)	17.26 ± 7.88(35)
SSCS	10.82 ± 9.57(33)	8.48 ± 7.11(27)	16.40 ± 10.18(20)	22.63 ± 10.24(38)	20.21 ± 9.46(34)	20.65 ± 10.04(37)
BRS	3.63 ± 0.67(33)	3.51 ± 0.68(32)	2.93 ± 0.72(20)	3.23 ± 0.81(39)	3.31 ± 0.80(36)	3.28 ± 0.75(37)
Bayer ADL	8.11 ± 1.76(39)	7.59 ± 2.21(36)	8.30 ± 2.00(37)	−	−	−
Barthel Index	74.36 ± 22.04(39)	75.14 ± 21.33(35)	67.92 ± 23.92(36)	−	−	−
NPI sum	29.05 ± 18.14(40)	23.60 ± 15.77(37)	26.60 ± 18.48(37)	−	−	−
NPI burden	−	−	−	16.21 ± 9.96(34)	14.00 ± 10.00(32)	16.90 ± 12.84(30)

*PwD, people living with dementia; IC, informal caregiver; GDS-15, Geriatric Depression Scale; PSS-10, Perceived Stress Scale; SSCS, Screening Scale for Chronic Stress taken from the Trier Inventory for the Assessment of Chronic Stress; BRS, Brief Resilience Scale; ADL, activities of daily living; NPI, neuropsychiatric inventory; X, mean; SD, standard deviation; n, sample size.*

Using MLM, we tested whether the scores on each of the psychometric test battery changed over time depending on the person (IC vs. PwD) and relationship (spousal vs. child–parent dyad) while controlling for gender. However, we found no significant main effect of Time in any of the models. Only in the models concerning the SSCS and BRS did a significant main effect of the Person (SSCS) and Relationship (BRS) emerge. These main effects can be interpreted such that PwD reported lower SSCS sum scores than did ICs, and that child–parent dyads reported higher resilience than did spousal dyads. All results can be found in Table 2.

**TABLE 2:** Results of linear mixed models predicting changes in psychometric test battery depending on time (admission, discharge, and follow-up), person (PwD and IC), and relationship (spousal dyad and child–parent dyad).

	GDS-15	PSS-10	SSCS	BRS	Bayer ADL	Barthel-Index	NPI sum	NPI burden
								
	Estimate (*SE*), *t*	Estimate (*SE*), *t*	Estimate (*SE*), *t*	Estimate (*SE*), *t*	Estimate (*SE*), *t*	Estimate (*SE*), *t*	Estimate (*SE*), *t*	Estimate (*SE*), *t*
**Base model**								
BIC	1029.786	1320.499	1337.736	416.235	826.643	1877.921	1896.713	1401.411
Intercept	4.51 (0.34), 13.464***	16.99 (0.69), 24.667***	16.85 (1.17), 14.456***	3.35 (0.07), 45.013***	8.01 (0.20), 39.823***	71.16 (2.37), 29.986***	26.74 (1.67), 16.046***	15.52 (1.09), 14.300***
**Full model**								
BIC	971.693	1234.869	1234.055	400.832	809.334	1782.340	1803.133	1336.187
Intercept	4.29 (1.13), 3.797***	17.18 (2.37), 7.250***	20.41 (3.52), 5.802***	2.90 (0.25), 11.555***	8.05 (0.36), 22.198***	−74.52 (4.76), 15.657***	22.02 (3.25), 6.770***	15.17 (1.93), 7.876***
Time	0.48 (0.37), 1.287	0.46 (0.95),0.490	−0.28 (1.19), −0.238	0.13 (0.10), 1.251	0.06 (0.13),0.443	−3.02 (1.70), −1.776	1.86 (1.40), 1.324	0.98 (0.91), 1.077
Gender	0.45 (1.27),0.353	2.15 (2.67),0.805	1.42 (3.97),0.359	0.28 (0.28),0.982	0.00 (0.51),0.001	1.79 (6.65),0.269	−0.24 (4.52), −0.053	−0.36 (2.71), −0.133
Person	−0.07 (1.25), −0.057	−3.49 (2.64), −1.323	−8.48 (3.97), −2.133*	0.49 (0.28), 1.749	–	–	–	–
Relationship	−2.35 (1.55), −1.519	0.91 (3.31),0.276	2.09 (4.99),0.418	0.80 (0.35), 2.242*	−1.34 (0.71), −1.887	9.68 (8.99), 1.077	4.96 (6.07),0.818	−4.84 (3.64), −1.329
Time*Gender	−0.28 (0.39), −0.721	−0.38 (1.01), −0.374	0.85 (1.26),0.678	−0.09 (0.11), −0.791	−0.00 (0.17), −0.008	−1.83 (2.15), −0.854	0.25 (1.78),0.139	0.77 (1.17),0.657
Gender*Relationship	1.28 (1.53),0.836	−2.25 (3.26), −0.690	−1.58 (4.98), −0.318	−0.46 (0.35), −1.300	1.16 (0.87), 1.335	−12.62 (10.77), −1.172	10.27 (7.19), 1.428	5.90 (4.48), 1.318
Time*Relationship	−0.46 (0.36), −1.280	−2.29 (0.91), −2.503*	−2.97 (1.12), −2.642**	−0.14 (0.10), −1.440	0.03 (0.17),0.154	−0.45 (2.11), −0.215	−7.87 (1.77), −4.460***	−3.19 (1.22), −2.609*
Time*Person	0.54 (0.35), 1.535	2.55 (0.93), 2.726**	2.98 (1.19), 2.504*	−0.32 (0.10), − 3.126**	–	–	–	–
Gender*Person	−3.80 (1.50), −2.536*	−5.51 (3.17), −1.738	−7.39 (4.88), −1.512	0.54 (0.34), 1.570	–	–	–	–
Relationship*Person	5.24 (1.46), 3.600***	6.41 (3.09), 2.074*	2.53 (4.76),0.531	−0.72 (0.34), −2.133*	–	–	–	–

BIC, Bayesian Information Criterion; PwD, people living with dementia; IC, informal caregiver; GDS-15, Geriatric Depression Scale; PSS-10, Perceived Stress Scale; BRS, Brief Resilience Scale; ADL, activities of daily living; NPI, neuropsychiatric inventory; Gender: 0 male, 1 female; Person: 0 IC, 1 PwD; Relationship: 0 spousal dyad, 1 child–parent dyad; SE, standard error; t, t-value; ***p < 0.001, **p < 0.01, *p < 0.05, fixed effects are reported here.

Furthermore, a series of interaction terms were significant. The Time*Relationship interaction was significant regarding the NPI sum score, NPI burden, the PSS-10, and the SSCS. Graphical illustration of these interactions show an effect of Time for ICs of child–parent dyads, with the sum scores on all of these scales decreasing from admission to follow-up, representing decreases in BPSD and associated burden as well as reduced subjective stress levels. Furthermore, significant Time*Person interactions were found concerning the PSS-10, SSCS, and BRS in that that perceived stress levels and chronic stress increased in PwD over time while resilience decreased. These figures remained stable from admission to discharge, and changed particularly from discharge to follow-up. Furthermore, the interaction term Relationship*Person was significant concerning the GDS-15, PSS-10, and BRS, showing that PwD in child–parent dyads had on average higher depression scores, higher perceived stress, and lower resilience compared to PwD in spousal dyads.

In sum, when evaluating the treatment trajectories based on subjective data there were no significant changes over time in scores concerning the psychometric test battery in the total sample. However, there were differential effects as PwD reported lower chronic stress than ICs while perceived stress levels and chronic stress increased in PwD and resilience decreased over time. Further, there were significant differences between child–parent dyads and couples: child–parent dyads reported higher resilience than couple dyads. Furthermore, for child–parent dyads decreases in behavioral symptoms of dementia and associated burden and reduced subjective stress levels were found from admission to follow-up. At the same time, PwD in child–parent dyads had overall higher depression scores, higher perceived stress, and lower resilience.

A graphical illustration of all significant interaction terms can be found in Supplementary Material D.

### Physiological Stress Markers Over Time – Hair Cortisol Concentrations and Ambulatory Assessment Data

**FIGURE 2:**
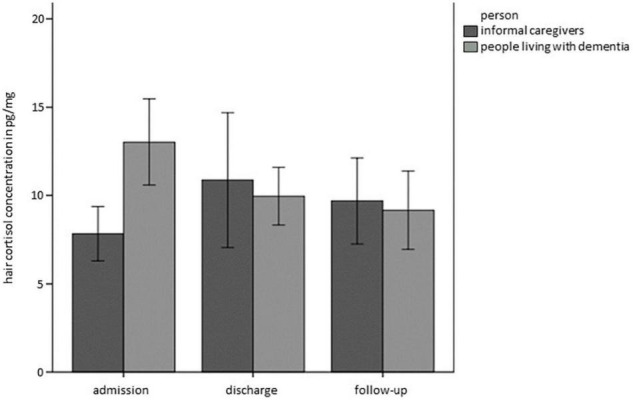
Hair cortisol concentrations (HCC) over time separately for PwD and ICs. Error bars represent standard error of the mean.

Concerning HCC, the full model showed a better fit than the base model (BIC 1406,537_*base model*_ vs. BIC_*full model*_ 1167,034). Whereas there was no main effect of Time, Relationship or Person, there was a significant interaction of Time*Person (*Estimate* = −6.34, *SE* = 2.83, *t* = −2.241, *p* < 0.05), as graphically depicted in Figure 2. This interaction term can be interpreted such that PwD showed decreases in HCC over time, while there were no significant differences in HCC over time in ICs.

Data from the ambulatory assessment are descriptively presented in Table 3. A visual inspection of the data shows normal diurnal rhythms of IC and PwD regarding cortisol secretion as both show typical cortisol awakening response (CAR) at the beginning and end of day clinic treatment. ICs further showed a typical awakening response in alpha-amylase at both time points. In contrast, PwD only showed the typical decline in sAA after awakening only at the beginning of day clinic treatment, but not at discharge from the day clinic.

**TABLE 3:** Ambulatory assessment data.

	Awakening	+30 min	10 am	2 pm	6 pm	9 pm
						
	*X* ± *SD* (*n*)	*X* ± *SD* (*n*)	*X* ± *SD* (*n*)	*X* ± *SD* (*n*)	*X* ± *SD* (*n*)	*X* ± *SD* (*n*)
**IC**						
**Admission**						
VAS	21.74 ± 22.21 (54)	27.89 ± 26.64 (53)	25.56 ± 21.88 (46)	27.80 ± 27.16 (50)	28.86 ± 27.13 (54)	19.33 ± 24.21 (52)
sCort	0.29 ± 0.35 (62)	0.47 ± 0.30 (61)	0.16 ± 0.14 (58)	0.13 ± 0.12 (55)	0.07 ± 0.07 (59)	0.06 ± 0.06 (57)
sAA	217.30 ± 268-03 (54)	132.62 ± 151.66 (54)	224.62 ± 216.40 (59)	220.63 ± 202.08 (55)	249.85 ± 216.96 (58)	277.53 ± 254.74 (59)
**Discharge**						
VAS	18.83 ± 21.51 (41)	22.07 ± 21.85 (42)	30.11 ± 26.61 (39)	23.15 ± 23.34 (38)	27.70 ± 26.87 (38)	21.53 ± 22.27 (36)
sCort	0.26 ± 0.23 (48)	0.50 ± 0.32 (51)	0.16 ± 0.12 (51)	0.14 ± 0.18 (50)	0.07 ± 0.05 (48)	0.05 ± 0.04 (46)
sAA	299.03 ± 355.95 (43)	145.91 ± 206.05 (44)	213.28 ± 240.74 (47)	319.09 ± 273.46 (46)	274.33 ± 286.68 (47)	242.46 ± 208.73 (47)
**PwD**						
**Admission**						
VAS	16.68 ± 26.56 (28)	15.67 ± 15.73 (27)	14.69 ± 19.06 (48)	13.58 ± 19.93 (50)	18.04 ± 19.29 (28)	19.52 ± 22.10 (27)
sCort	0.47 ± 0.38 (34)	0.56 ± 0.36 (37)	0.22 ± 0.15 (55)	0.20 ± 0.20 (56)	0.18 ± 0.33 (36)	0.11 ± 0.12 (35)
sAA	172.63 ± 253.42 (31)	153.41 ± 218.70 (33)	208.39 ± 211.53 (58)	303.27 ± 291.94 (57)	260.45 ± 259.00 (36)	266.08 ± 285.92 (35)
**Discharge**						
VAS	14.65 ± 21.86 (26)	23.54 ± 25.24 (27)	18.37 ± 26.47 (47)	17.67 ± 25.83 (44)	21.18 ± 23.37 (28)	20.19 ± 25.40 (25)
sCort	0.33 ± 0.18 (31)	0.41 ± 0.24 (34)	0.21 ± 0.14 (55)	0.18 ± 0.17 (54)	0.09 ± 0.08 (33)	0.10 ± 0.08 (33)
sAA	134.63 ± 212.70 (27)	175.50 ± 261.22 (29)	252.56 ± 268.82 (54)	316.90 ± 345.33 (52)	255.97 ± 251.77 (30)	200.15 ± 199.09 (31)

*PwD, people living with dementia; IC, informal caregiver; VAS, visual analog scale on subjective, momentary stress ranging from 0 (not at all) to 100 (completely); sCort, salivary cortisol secretion in μg/dl; sAA, salivary alpha-amylase activity in U/ml; X, mean; SD, standard deviation; n, sample size.*

The models of the MLMs predicting differences in summary indicators concerning alpha-amylase did not deliver results as the models had too many missing values. Models regarding summary indicators of cortisol secretion (CAR and AUCg) and momentary subjective stress (VAS) did not yield any significant differences. The complete statistics concerning these models can be found in the Supplementary Material E.

In sum, complementing the findings on subjective ratings with physiological stress markers, discrepancies between subjective and physiological markers of stress emerged in PwD: we found significant differences in HCC, with PwD showing decreases in HCC over time while reporting subjectively increased chronic stress over time. From a descriptive perspective, moreover, HCC at baseline was higher in PwD than in ICs, although PwD subjectively reported lower chronic stress than did ICs. Data from the ambulatory assessment revealed characteristic diurnal profiles for both PwD and ICs, however, there were no significant differences in summary indicators over time.

## Discussion

### Summary

We set out to evaluate the feasibility of a psychobiological evaluation of day clinic treatment in PwD and their ICs to evaluate treatment trajectories considering both the day clinic context and the daily life of the dyads. The feasibility of psychobiological measures varied between the different outcome variables. Whereas all participants were willing to provide subjective reports, not all participants were willing or had the possibility to collect physiological stress markers. Concerning HCC, 11.25% of the sample did not provide hair samples and concerning saliva samples as part of the ambulatory assessment, 20.00% of the sample did not provide saliva samples at all. However, when considering the sub-samples that provided physiological stress markers, completion rates were comparable between paper-and-pencil questionnaires (71–100%) and HCC (77–90%), but lower in saliva samples (75–78%) making HCC a particular promising physiological stress marker in PwD and IC. Validity of self-reports in PwD was high in for the GDS-15, PSS-10, and SSCS and only moderate in BRS.

Concerning treatment trajectories we found discrepancies between subjective and physiological markers of stress in PwD with decreases in HCC over time while reporting subjectively increased chronic stress over time. Although feasibility was high in subjective reports, PwD might use different time perspectives than described in the instruction of the respective scales. Despite the challenges in collecting physiological stress markers in this particular population, the discrepancy between subjective and physiological markers emphasizes the added value of assessing physiological markers of stress to complement the evaluation of PwDs’ and ICs’ stress experience. Thus, physiological stress markers hold the potential to inform diagnosis and treatment trajectories in both PwD and ICs.

### Feasibility and Added Value of Psychobiological Stress Markers

The assessment of psychobiological stress markers in PwD was feasible. Although Arsenault-Lapierre et al. (2012) reported that anosognosia in PwD was correlated with anosognosia for perceived stress, the validity of self-reports was high for subjective stress measures in PwD. Nevertheless, it has to be critically discussed, that we used statistical measures to evaluate the validity of self-reports in PwD, whereas Arsenault-Lapierre et al. (2012) considered the discrepancy between self-report and caregiver-based ratings as a measure for anosognosia. This raises the question of whether it is possible to rate perceived stress levels externally. Regarding the validity of informant-based and self-reported ratings, Kaiser et al. (2022) found a high discrepancy between observer- and patient-reported outcomes when evaluating the success of depression treatment, thus emphasizing the need to directly include the perspective of the patient. As the potential to include the patient’s perspective is particularly limited in the case of a dementia diagnosis, our data support the notion of adding physiological markers to complement the patient’s perspective. Likewise, Arsenault-Lapierre et al. (2012) found no association between anosognosia and cortisol levels, leading to the assumption that physiological stress markers are particularly robust in PwD. In the present study, we also found a higher completion rate for HCC compared to the ambulatory assessment data, possibly because HCC was assessed at three time points by study personnel, in comparison to participants being required to collect saliva samples independently, or with the assistance of ICs, over 24 different points in time in daily life as part of the ambulatory assessment. Both the assistance of study personnel and the day clinic context might have reduced burden for the participant in comparison to the ambulatory assessment at home. This makes HCC a particular promising physiological stress marker in PwD, as it can complement subjective reports on chronic stress levels while keeping participant burden to a minimum. This converges with Wright et al. (2015) who describe the potential of HCC as retrospective biological marker of stress in older adults.

Although perceived momentary stress levels should be complemented by ambulatory assessment data, the high number of missing values in our study limits the informative value in this regard. Interestingly, completeness was higher in spousal dyads than child–parent dyads. From this, it might be recommended that PwD need assistance and support in collecting saliva samples, which is probably more likely to be the case with spouses than with children due to physical proximity. Another way to increase feasibility might be the use of electronic devices that emit prompts to remind participants to complete the assessments. However, we decided against the use of technical equipment in order not to overburden the participants. Interestingly, whereas almost 30% of the sample decided not to provide saliva samples, the remaining participants completed around 80% of saliva samples. This might be interpreted as suggesting that higher completeness and feasibility are associated with more selective study samples. Thus, one might need to weigh the specificity of a sample against its generalizability. Further, larger study samples are necessary to detect effects in daily life by means of ambulatory assessment data.

Comparing the feasibility of our psychobiological assessment to the existing literature, different settings and populations need to be kept in mind. Studies with high numbers of missing values were most often set in nursing homes addressing agitated residents with dementia: Pu et al. (2020) analyzed saliva samples from 8 out of 43 PwD, Kwan et al. (2016) analyzed 161 out of 360 saliva samples. Although these studies were set in a controlled setting where study personnel was available to assist in the collection of saliva samples, high amounts of missing values were found due to refusal of PwD to collect saliva samples, cognitive impairments, or inadequate saliva volume. In contrast, missing values in PwD from residential age care without agitation and home dwelling PwD were lower compared to agitated PwD in nursing homes, as D’Cunha (2019) was able to analyze saliva samples from 22 out of 25 PwD. These comparisons stress the fact that the feasibility of saliva samples is closely linked to the severity of dementia. Likewise, in our study missing values in saliva samples increased with dementia severity. On the other hand, missing values in HCC were not related to disease severity but depressive symptoms. This emphasized the potential of HCC in patients with severe dementia to complement informant-based ratings with physiological data on stress markers.

In terms of content, we found further evidence for the added value of psychobiological markers in PwD. In a recent study by our work group (Wuttke-Linnemann et al., 2022), we also found discrepancies between subjective and physiological markers of stress concerning the evaluation of a home-based dyadic psychosocial intervention for patients with mild to moderate dementia and their ICs. The discrepancy between HCC and subjective stress measures found in this study supports the notion that physiological stress markers complement subjective measures in all stages of the disease. This is in line with Stalder et al. (2017) who reports no consistent correlations among HCC and subjective stress measures, specifically in chronically stressed populations. Various reasons can be assumed for this, and in particular in the context of dementia care, this discrepancy might have important implications. Based on our data, we cannot answer whether this means that the subjective data are less valid than assumed, but we can use disease-specific knowledge to generate hypotheses for future studies. In particular, HCC was higher in PwD than in ICs at admission to the clinic, even though PwD reported lower subjective chronic stress. This could be interpreted as suggesting that PwD tend to downplay their symptoms in order to avoid attracting attention, particularly in the context of a clinic admission, which brings about unwanted disruptions to one’s daily routine, thus implying motivational reasons for this discrepancy. Another explanation might concern the cognitive deficits. As dementia involves memory impairments, it might be the case that PwD rate their momentary stress level as they cannot accurately remember how their stress level has been within the last 4 weeks. This would explain why validity was statistically high although PwD might have used a different time scale when answering the items on subjective chronic stress. Accordingly, this explanation would allude to cognitive deficits and reduced informative value of self-reports in PwD. Furthermore, HCC decreased over time in PwD despite the fact that subjective stress reports increased. Again, this might suggest that conceptually, self-reports do not match physiological stress markers in PwD very well, as they assess different stress constructs and different time scales.

### Day Clinic Treatment Trajectories in People Living With Dementia and Informal Caregivers

Overall, the assessed variables remained stable over time, with the exception of subjective stress levels, which increased in PwD over time while resilience decreased. However, this does not imply that day clinic treatment does not work for PwD. Our previous study also found differences over time in dementia-specific assessment instruments (Linnemann et al., 2018) as well as no differences in treatment trajectories between day clinic and inpatient settings, rendering day clinic treatment a valid treatment alternative. In the context of a neurodegenerative disorder like dementia, stabilizing effects are considered to be worthwhile as well (Linnemann et al., 2018). Indeed, the finding that autonomy and BPSD did not worsen over a time period of 6 months can be seen as a success.

Nevertheless, in the present study, treatment trajectories differed according to dyad type, with child–parent dyads benefiting more from day clinic treatment than spousal dyads. In fact, child–parent dyads even showed a reduction in BPSD and associated burden and stress from admission to follow-up. Furthermore, PwD from child–parent dyads were more affected by depression and increased stress. This is reminiscent of the results of the aforementioned previous study (Linnemann et al., 2018), which reported that caregivers of PwD from a day clinic were older and thus more often represented spousal than child–parent dyads. The spousal caregivers were also more physically and psychologically impaired, both at baseline and at follow-up. Our results thus add the insight that day clinic treatment might be more feasible and effective in child–parent dyads than in spousal dyads. We can only speculate on the underlying reasons for this finding. One explanation might be that spousal dyads most often live together and share the majority of daily life. In line with this, Kürten et al. (2021) found that the time ICs need to supervise the PwD predicted depressiveness in ICs. This might explain the differences in treatment trajectories that we found between child–parent and spousal dyads, as spousal dyads may be required to spend more time on caregiver duties, if residing in the same household as the care recipient. Accordingly, spousal caregivers might thus experience day clinic treatment as less relieving compared to inpatient treatment, as the morning and evening/night-time hours still have to be covered by the ICs.

### Limitations

Although the study protocol combined subjective and physiological markers of stress repeatedly over time in a difficult-to-reach vulnerable population, certain methodological issues warrant critical attention: First of all, the assessments at admission and discharge were scheduled in the days *after* admission and the days *prior* to discharge. Thus, we cannot draw any conclusions about how psychobiological measures might have varied *before* admission and *after* discharge. It is possible that the mere admission to the day clinic already relieved certain symptoms and that the assessment of our variables in the week before admission might have led to a different profile. Furthermore, as we collected data at discharge, it is possible that the imminent discharge brought about an exaggeration of possible effects of treatment. Moreover, we do not know how the transition back to the home environment affected the dyads. These are important research questions that should be addressed in future studies. In addition, the higher ecological validity of the ambulatory assessment data is accompanied by lower internal validity. Specifically, as the PwD needed assistance from their ICs when collecting the saliva samples, it remains unclear whether this affected the dyad. In particular, we do not know whether the ICs perceived assisting in collecting saliva samples from the PwD, while also collecting their own saliva samples, as stressful. Future investigations need to evaluate methods to perform ambulatory assessment in PwD and their ICs in a non-intrusive manner. Furthermore, our study sample was selective, with an overrepresentation of female ICs, thus limiting conclusions on male ICs. However, this gender distribution is often found in studies, as ICs of PwD are predominantly female. Finally, the study was exploratory in nature, with no control group, thus preventing conclusions on causality. Future studies are necessary to compare characteristics, treatment trajectories, and treatment effects between day clinic and inpatient settings in a randomized controlled design.

### Conclusion

The feasibility of the psychobiological evaluation of the day clinic treatment varied according to the different stress measures. The highest feasibility was found for subjective stress measures and hair cortisol concentrations. Despite cognitive deficits, subjective stress reports showed high validity although due to the memory deficits in PwD they may represent different time perspective than stated in the instruction. Ambulatory assessment data showed many missing values, with 20% of the sample unwilling or unable to collect saliva samples. This high number of missing values limits the informative value regarding effects in daily life. However, when dyads did decide to collect saliva samples, they showed high rates of completion. Thus, in selective samples the assessment of salivary stress measures was feasible, whereas in more heterogenous samples the collection of hair cortisol concentrations might be preferred as less active engagement of the participant is necessary. There was a discrepancy between subjective and physiological stress markers in PwD, emphasizing the fact that physiological markers complement subjective reports in a meaningful way. Treatment trajectories revealed stabilizing effects for PwD over time, but differed between spousal and child–parent dyads, with child–parent dyads generally appearing to benefit more from day clinic treatment compared to spousal dyads. Overall, the psychobiological evaluation of day clinic treatment was feasible for PwD and ICs, and future studies need to corroborate these findings in larger samples. Physiological stress markers, particularly hair cortisol, hold the potential to become an objective marker of stress that is relevant in diagnosis and treatment of dementia both from a preventive as well as disease modifying perspective.

## Data Availability Statement

The raw data supporting the conclusions of this article will be made available by the authors, without undue reservation.

## Ethics Statement

The studies involving human participants were reviewed and approved by the Ethics Committees of the Landesärztekammer Bayern (as the day clinic was situated in Munich) and the Landesärztekammer Rheinland-Pfalz (as the evaluation was coordinated in Mainz). The patients/participants provided their written informed consent to participate in this study.

## Author Contributions

AW-L and AF conceived the study and were in charge of overall direction, coordination, and planning. TB assisted in collecting the data and entered the data into a database. SP prepared the data for data analysis. SP and AW-L performed the data analysis. KE supervised the analysis of salivary markers. UN and NS supervised the analysis of hair cortisol samples. AW-L wrote the first draft of the manuscript with support from KG and SP. All authors provided critical feedback and helped to shape the manuscript.

## Conflict of Interest

The authors declare that the research was conducted in the absence of any commercial or financial relationships that could be construed as a potential conflict of interest.

## Publisher’s Note

All claims expressed in this article are solely those of the authors and do not necessarily represent those of their affiliated organizations, or those of the publisher, the editors and the reviewers. Any product that may be evaluated in this article, or claim that may be made by its manufacturer, is not guaranteed or endorsed by the publisher.
