# Supplementary material for: Psychobiological Evaluation of Day Clinic Treatment for People Living With Dementia – Feasibility and Pilot Analyses
Source: Front Aging Neurosci. 2022 Jun 30;14:866437. doi: 10.3389/fnagi.2022.866437 (PMC9279127; doi:10.3389/fnagi.2022.866437)
Supplement: Supplementary file 1 [file Table_1.docx]

**Supplementary Material A**

Summary of models in which a random effect of the variable Time improved model fit

| Random Effects (Variances) | Bayer-ADL score  Estimate (SE) Wald z | NPI Burden score  Estimate (SE) Wald z | Hair cortisol concentrations  Estimate (SE) Wald z |
| --- | --- | --- | --- |
| Level – 2 (between person) |  |  |  |
| Intercept ($\tau_{00})$ | 2.38*** (0.510) 4.665 | 71.76*** (14.464) 4.961 | 71.83* (31.68) 2.267 |
| Intercept, time covariance ($\tau_{10})$ | 0.290 (0.206) 1.407 | -2.61 (5.268) -0.496 | -20.07* (9.82) -2.045 |
| Time ($\tau_{11})$ |  | 12.34 *** (3.561) 3.466 |  |
|  |  |  |  |
| Level-1 (within-person) |  |  |  |
| Residual ($\sigma_{\varepsilon}^{2}$) | 0.94*** (0.135) 6.927 | 20.18*** (3.953) 5.105 | 103.06*** (20.11) 5.125 |

Annotations: Bayer-ADL: Activities of Daily Living, NPI: Neuropsychiatric Inventory; SE: standard error, * p < .05, ** p < .01, *** p <.001
