# Supplementary material for: Psychobiological Evaluation of Day Clinic Treatment for People Living With Dementia – Feasibility and Pilot Analyses
Source: Front Aging Neurosci. 2022 Jun 30;14:866437. doi: 10.3389/fnagi.2022.866437 (PMC9279127; doi:10.3389/fnagi.2022.866437)
Supplement: Supplementary file 2 [file Table_2.docx]

|  | CAR (0/1) | sCort (0/1) | sAA (0/1) | HCC (0/1 | Person | Time | MMSE | NPI | GDS-15 | Gender | Relationship |
| --- | --- | --- | --- | --- | --- | --- | --- | --- | --- | --- | --- |
| CAR (0/1) | 1 | 0.747** | 0.699** | 0.115* | -0.180** | -0.526** | 0.178** | -0.071 | -0.140* | -0.081 | -0.228** |
| sCort (0/1) | 0.747** | 1 | 0.923** | 0.087 | -0.220** | -0.399** | 0.212** | -0.084 | -0.195** | 0.008 | -0.189** |
| sAA (0/1) | 0.699** | 0.923** | 1 | 0.06 | -0.212** | -0.377** | 0.181** | -0.044 | -0.133* | -0.02 | -0.162** |
| HCC (0/1) | 0.115* | 0.087 | 0.060 | 1 | 0.047 | -0.104 | 0.001 | -0.101 | -0.195** | 0.112* | -0.082 |

**Supplementary Material B**

Correlations among occurrence of missing values in physiological stress markers (CAR, sCort, sAA, HCC) and predictor variables (person, time, MMSE sum score, NPI sum score, GDS-15, gender and relationship)

*Annotations: 0/1: 0 = missing value, 1 = value present; CAR: cortisol awakening response, sCort: saliva sample for measurement of salivary cortisol, sAA: saliva sample for measurement of alpha-amylase, HCC: hair cortisol sample, Person: 0 = informal caregiver, 1 = people living with dementia; MMSE: sum score of the Mini Mental State Examination (lower figures indicate higher cognitive impairment), NPI: sum score Neuropsychiatric Inventory (higher scores indicate higher extent of behavioral and psychological symptoms of dementia), GDS-15: sum score of the Geriatric Depression score (higher scores indicating higher levels of depressive symptoms), Gender: 0 = female, 1 = male; Relationship: 0 Spousal dyad, 1 child-parent dyad, ** < .001, * < .01*
