# Supplementary material for: Psychobiological Evaluation of Day Clinic Treatment for People Living With Dementia – Feasibility and Pilot Analyses
Source: Front Aging Neurosci. 2022 Jun 30;14:866437. doi: 10.3389/fnagi.2022.866437 (PMC9279127; doi:10.3389/fnagi.2022.866437)
Supplement: Supplementary file 3 [file Table_3.docx]

**Supplementary Material C**

Results of 2x2 chi-square tests to test independence of missing values of the collected measures for each measurement time point.

|  | **Baseline**  **Χ^2^ (df) p** | **Discharge**  **Χ^2^ (df) p** | **Follow-Up**  **Χ^2^ (df) p** |
| --- | --- | --- | --- |
| GDS (0/1) x person | 6.49 (1)* | 0.11 (1) | 10.32 (1)** |
| PSS (0/1) x person | 1.05 (1) | 0.39 (1) | 10.31 (1)** |
| SCSS (0/1) x person | 3.13 (1) | 3.38 (1) | 17.64 (1)*** |
| BRS (0/1) x person | 5.00 (1)* | 1.57 (1) | 17.64 (1)*** |
|  |  |  |  |
| GDS (0/1) x CAR (0/1) | 0.05 (1) | 9.07 (1)** |  |
| GDS (0/1) x sAA (0/1) | 0.21 (1) | 3.19 (1) |  |
| GDS (0/1) x Cort (0/1) | 0.15 (1) | 3.47 (1) |  |
| GDS (0/1) x HCC (0/1) | 1.62 (1) | 18.87 (1)*** | 18.15 (1)*** |
| PSS (0/1) x CAR (0/1) | 2.53 (1) | 10.41 (1)*** |  |
| PSS (0/1) x sAA (0/1) | 2.96 (1) | 3.76 (1)* |  |
| PSS (0/1) x Cort (0/1) | 3.11 (1) | 4.08 (1)* |  |
| PSS (0/1) x HCC (0/1) | 0.07 (1) | 10.86 (1)** | 6.58 (1)* |
| SCSS (0/1) x CAR (0/1) | 3.68 (1) | 9.10 (1)** |  |
| SCSS (0/1) x sAA (0/1) | 1.52 (1) | 8.43 (1)** |  |
| SCSS (0/1) x Cort (0/1) | 1.71 (1) | 9.04 (1)** |  |
| SCSS (0/1) x HCC (0/1) | 1.13 (1) | 11.55 (1)** | 6.58 (1)* |
| BRS (0/1) x CAR (0/1) | 9.48 (1)** | 10.41 (1)*** |  |
| BRS (0/1) x sAA (0/1) | 3.03 (1) | 3.76 (1) * |  |
| BRS (0/1) x Cort (0/1) | 3.27 (1) | 4.08 (1) * |  |
| BRS (0/1) x HCC (0/1) | 1.70 (1) | 15.98 (1)*** | 6.58 (1)* |
|  |  |  |  |
| sAA (0/1) x HCC (0/1) | 0.05 (1) | 0.38 (1) |  |

*Annotations: 0/1: 0 = missing value, 1 = value present; Person: 0 = informal caregiver, 1 = people living with dementia; GDS: Geriatric Depression scale; PSS: Perceived Stress Scale; SCSS: Screening Scale for Chronic Stress; BRS: Brief Resilience Scale; CAR: cortisol awakening response; sCort: saliva sample for measurement of salivary cortisol; sAA: saliva sample for measurement of alpha-amylase; HCC: hair cortisol sample; *** < .001, ** < .01, * <.05, exact Fisher test is reported*
