# Supplementary material for: Psychobiological Evaluation of Day Clinic Treatment for People Living With Dementia – Feasibility and Pilot Analyses
Source: Front Aging Neurosci. 2022 Jun 30;14:866437. doi: 10.3389/fnagi.2022.866437 (PMC9279127; doi:10.3389/fnagi.2022.866437)

**Supplementary Material D**

Graphical Illustration of Significant Interaction Terms

- Joint graphical illustration of significant interaction terms Gender*Person and Relationship*Person concerning GDS-15 sum score


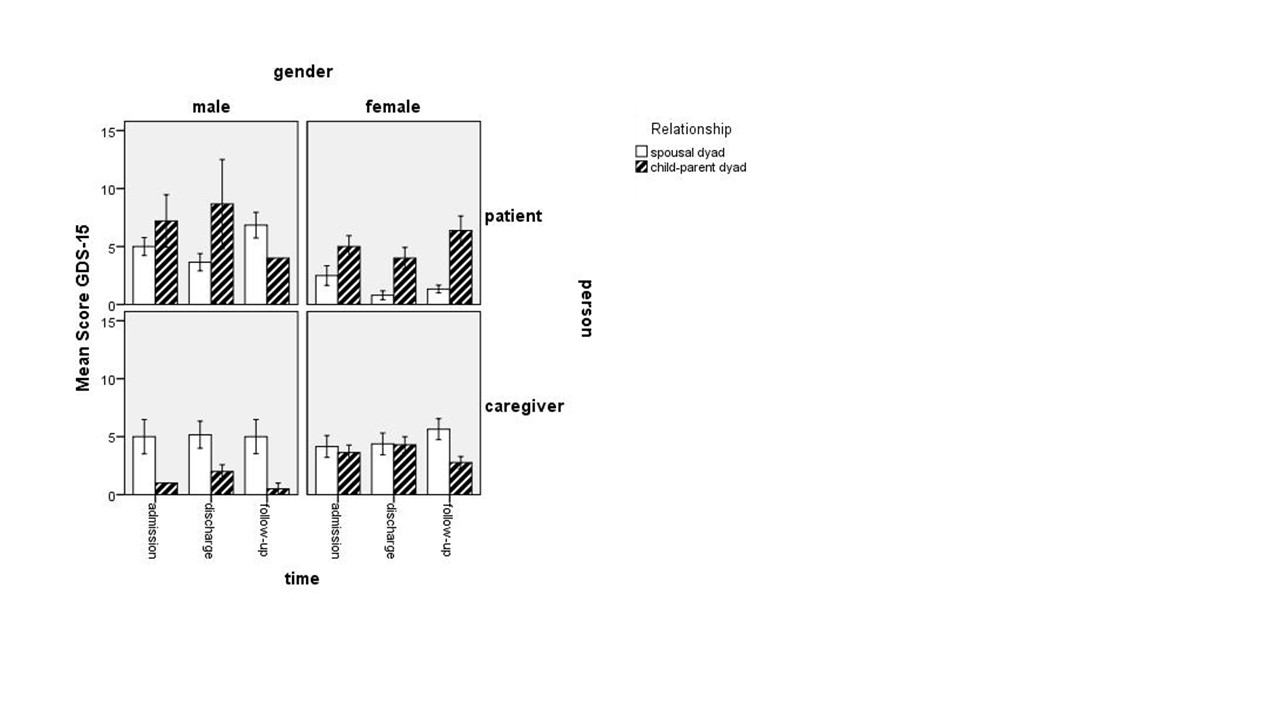


- Joint graphical illustration of significant interaction terms Time*Relationship, Time*Person, and Relationship*Person concerning PSS-10 sum score


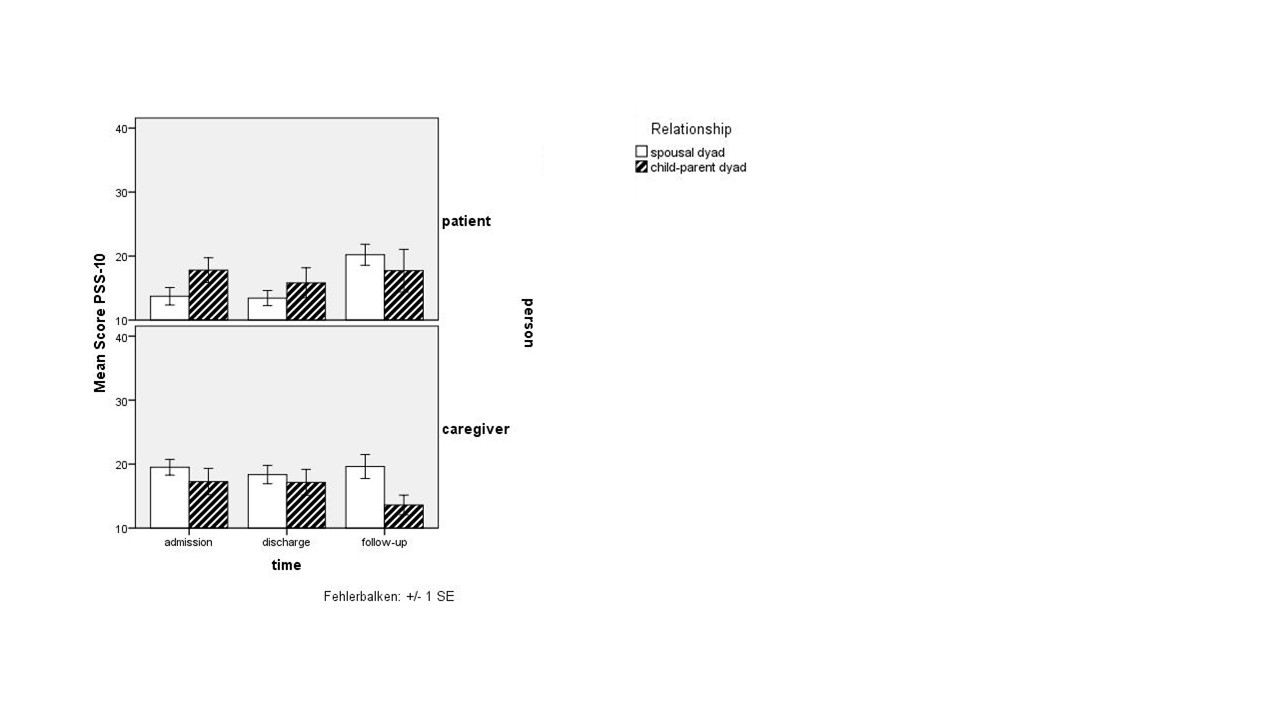


- Joint graphical illustration of significant interaction terms Time*Relationship and Time*Person concerning SSCS sum score


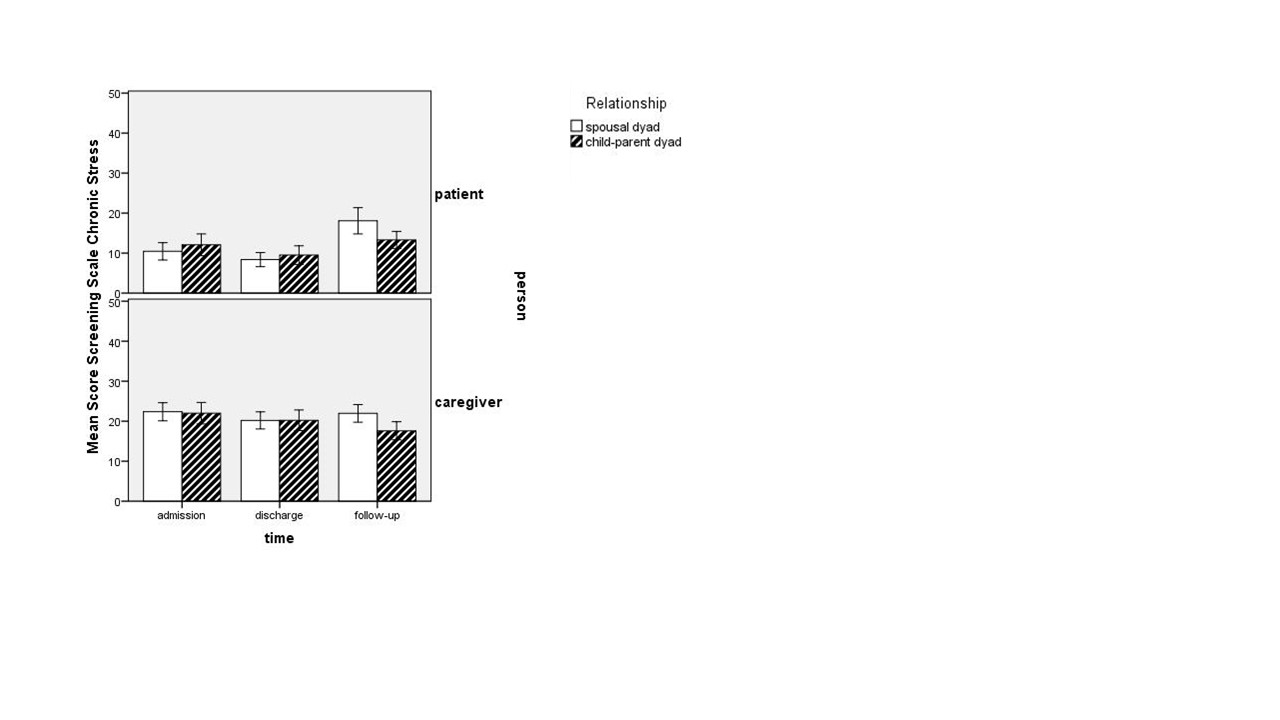


- Joint graphical illustration of significant interaction terms Relationship*Person and Time*Person concerning BRS score


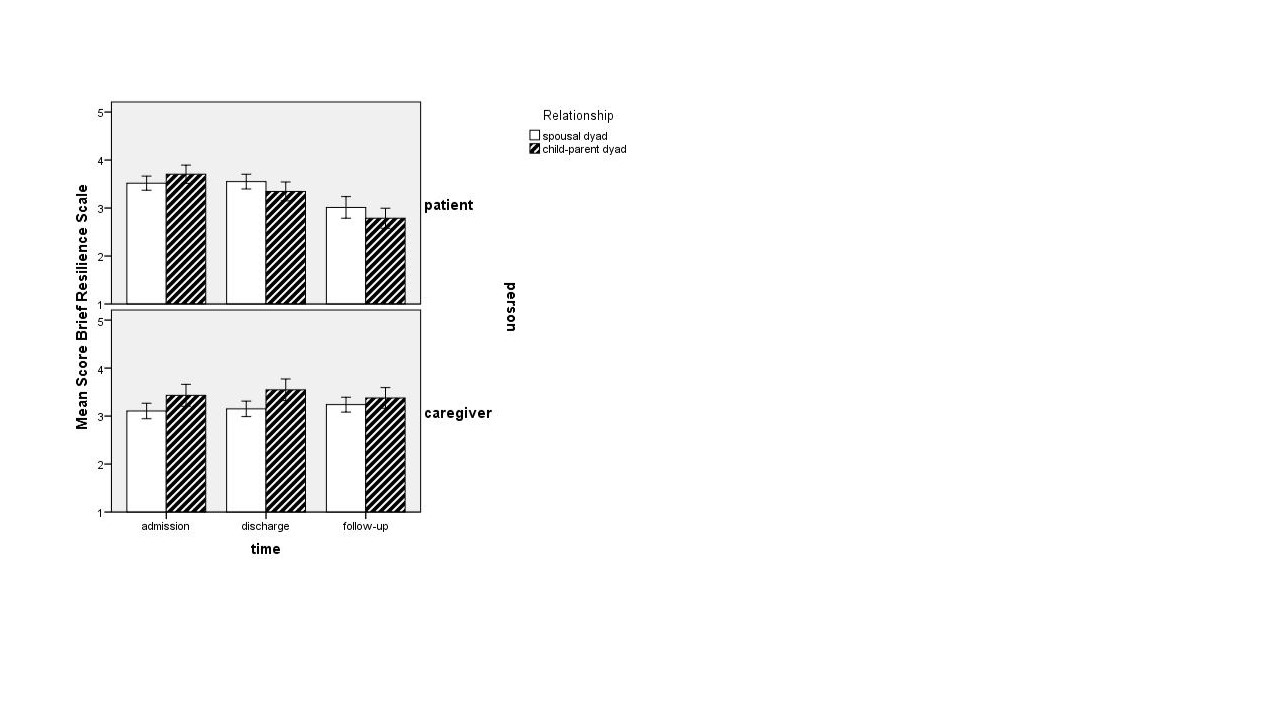


- Graphical illustration of significant interaction terms Time*Relationship concerning NPI sum score


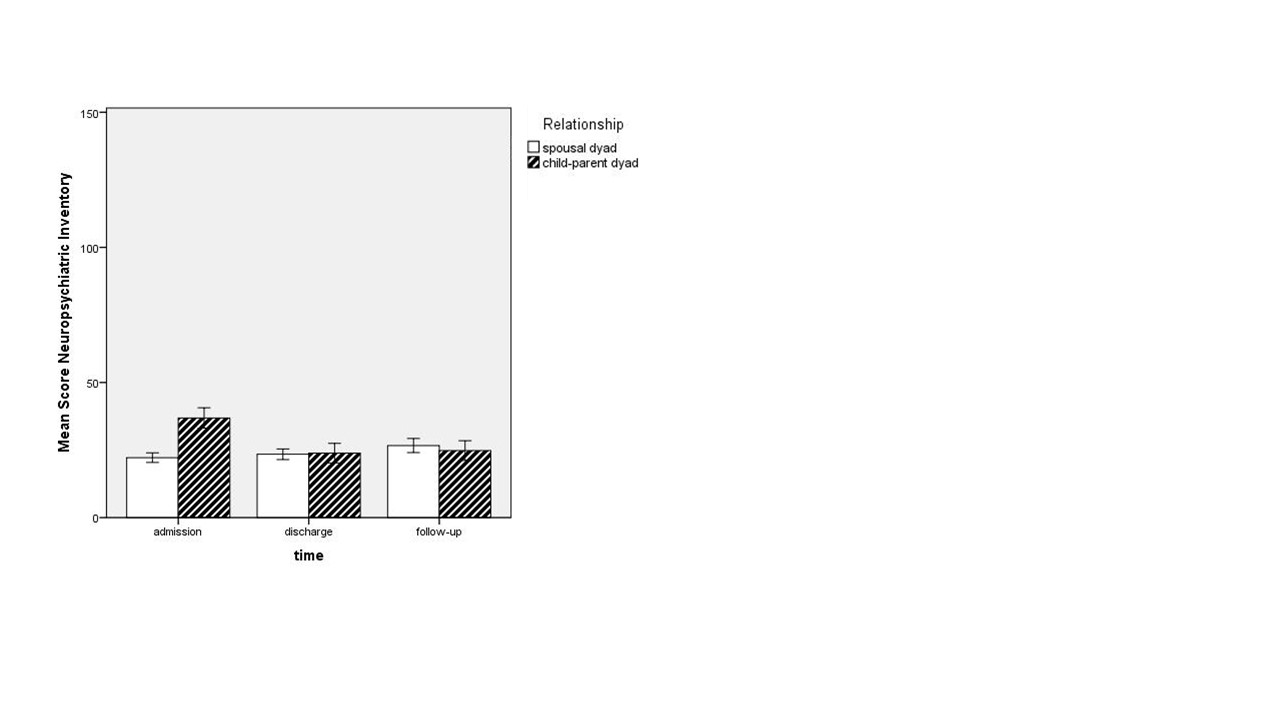


- Graphical illustration of significant interaction terms Time*Relationship concerning NPI burden score


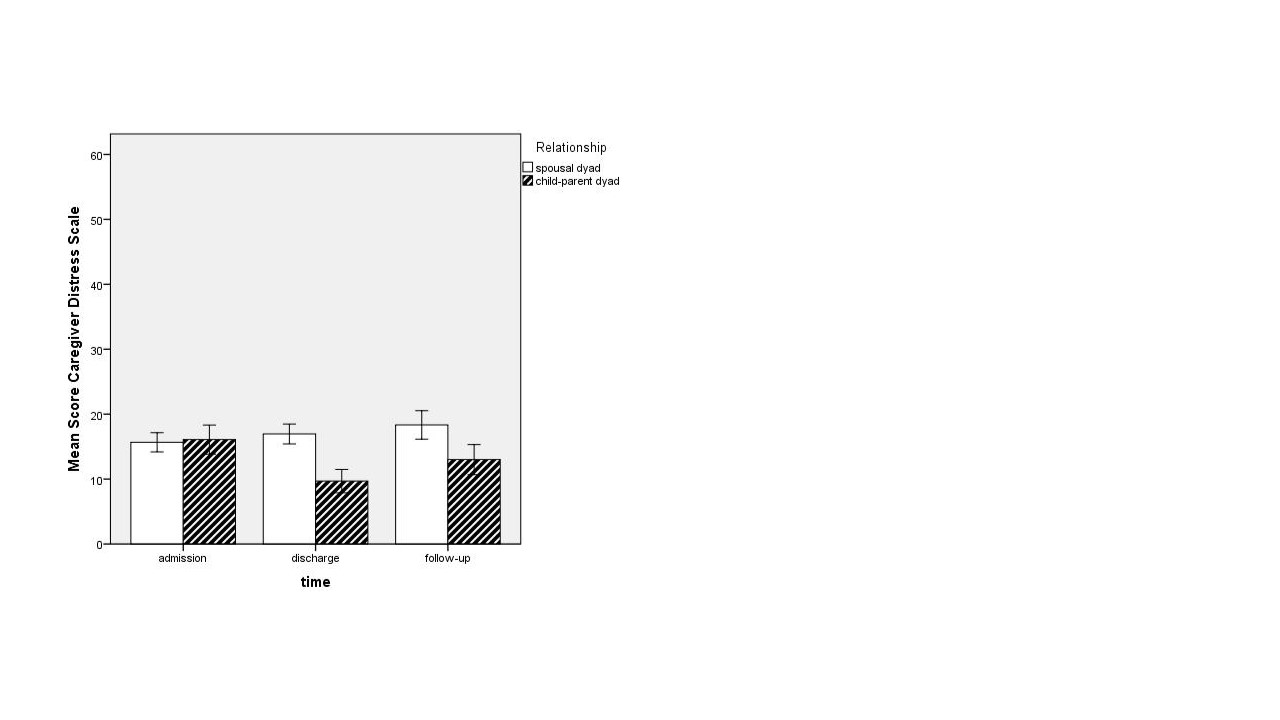

Supplement: Supplementary file 4 [file Table_4.docx]
