# Supplementary material for: Psychobiological Evaluation of Day Clinic Treatment for People Living With Dementia – Feasibility and Pilot Analyses
Source: Front Aging Neurosci. 2022 Jun 30;14:866437. doi: 10.3389/fnagi.2022.866437 (PMC9279127; doi:10.3389/fnagi.2022.866437)
Supplement: Supplementary file 5 [file Table_5.docx]

**Supplementary Material E**

Results of linear mixed models predicting changes in psychobiological stress markers depending on time (admission, discharge, follow-up), person (PwD, IC), and relationship (spousal dyad, child-parent dyad) controlling for Age, Gender and BMI

| Fixed effects | CAR  Estimate (SE), t | AUCg VAS  Estimate (SE), t | AUCg sCort  Estimate (SE), t | AUCg sAA  Estimate (SE), t |
| --- | --- | --- | --- | --- |
| Base model |  |  |  |  |
| BIC | 1162.973 | 1428.539 | 677.586 | - |
| Intercept | 114.72 (18.27), 6.280*** | 19030.37 (1968.80), 9.666*** | 158.22 (10.17), 15.562*** | - |
|  |  |  |  |  |
| Full model |  |  |  |  |
| BIC | 978.987 | 1374.226 | 493.907 | - |
| Intercept | 132.33 (264.08), 0.501 | 21145.62 (2736.73), 7.727*** | 288.01 (534.80), 0.539 | - |
| Time | 203.72 (128.07), 1.591 | -196.87 (3283.30),  -0.060 | -101.24 (184.52),  -0.549 | - |
| Person | -169.34 (260.40),  -0.650 | -7937.89 (4655.57),  -1.705 | -144.57 (399.28),  -0.362 | - |
| Age | - | - | -.23 (7.20), -0.032 | - |
| Gender | 69.04 (259.38), 0.266 | - | -247.37 (289.91),  -0.853 | - |
| BMI | -4.13 (10.75), -0.384 | - | -6.89 (18.99), -0.363 | - |
| Relationship | - | - | -3.41 (77.03), -0.044 | - |
| Time*Person | -96.42 (60.46),  -1.595 | 5204.95 (5383.70), 0.967 | -24.44 (35.01),  -0.698 | - |
| Time*Age | - | - | 1.39 (2.10), 0.661 | - |
| Time*Gender | -71.06 (59.11),  -1.202 | - | -52.30 (31.39),  -1.666 | - |
| Time*BMI | -3.53 (4.57), -0.772 | - | 0.75 (3.10), 0.241 | - |
| Time*Relationship | - | - | 82.71 (60.74), 1.362 | - |
| Person*Age | - | - | 0.00 (4.00), 0.001 | - |
| Person*Gender | -132.63 (83.12),  -1.596 | - | 25.64 (62.44), 0.411 | - |
| Person*BMI | 7.45 (10.36), 0.720 | - | 7.06 (7.57), 0.933 | - |
| Person*Relationship | - | - | -6.57 (96.25), -0.068 | - |
| Age*BMI | - | - | 0.03 (0.27), 0.098 | - |
| Age*gender | - | - | 2.17 (2.62), 0.828 | - |
| Gender*BMI | 3.24 (10.49), 0.309 | - | 4.24 (7.79), 0.544 | - |

Annotations: CAR: cortisol awakening response, AUCg: area-under-the-curve with respect to ground, VAS: visual analogue scale subjective momentary stress (higher figures indicate higher amounts of momentary stress), sCort: salivary cortisol secretion, sAA: salivary alpha-amylase activity, BIC: Bayesian Information Criterion, Gender: 0 = female, 1 = male, BMI: body-mass-index, Person: 0 = caregiver, 1 = people living with dementia, Time: 0 = admission, 1 = discharge, 2 = follow-up, Relationship: 0 = spousal dyads, 1 = child-parent dyads, SE: standard error, *** p <.001
